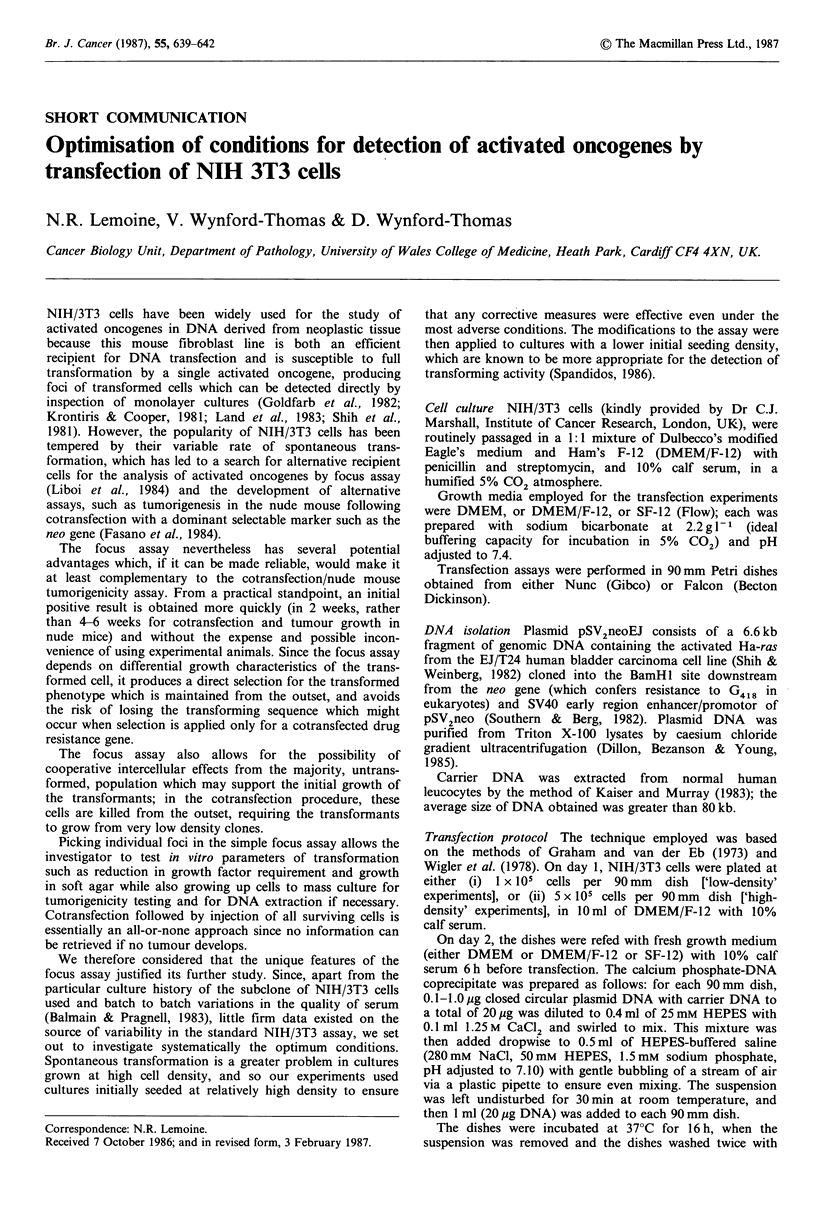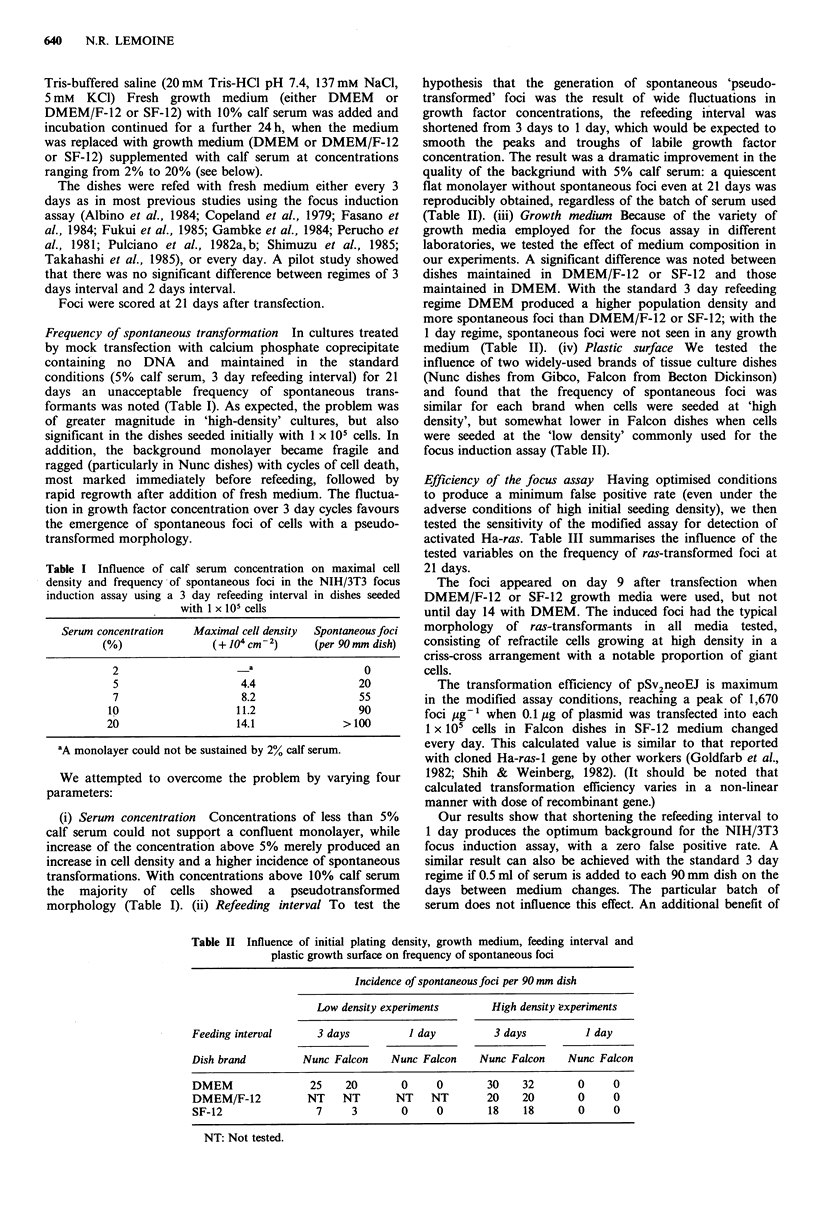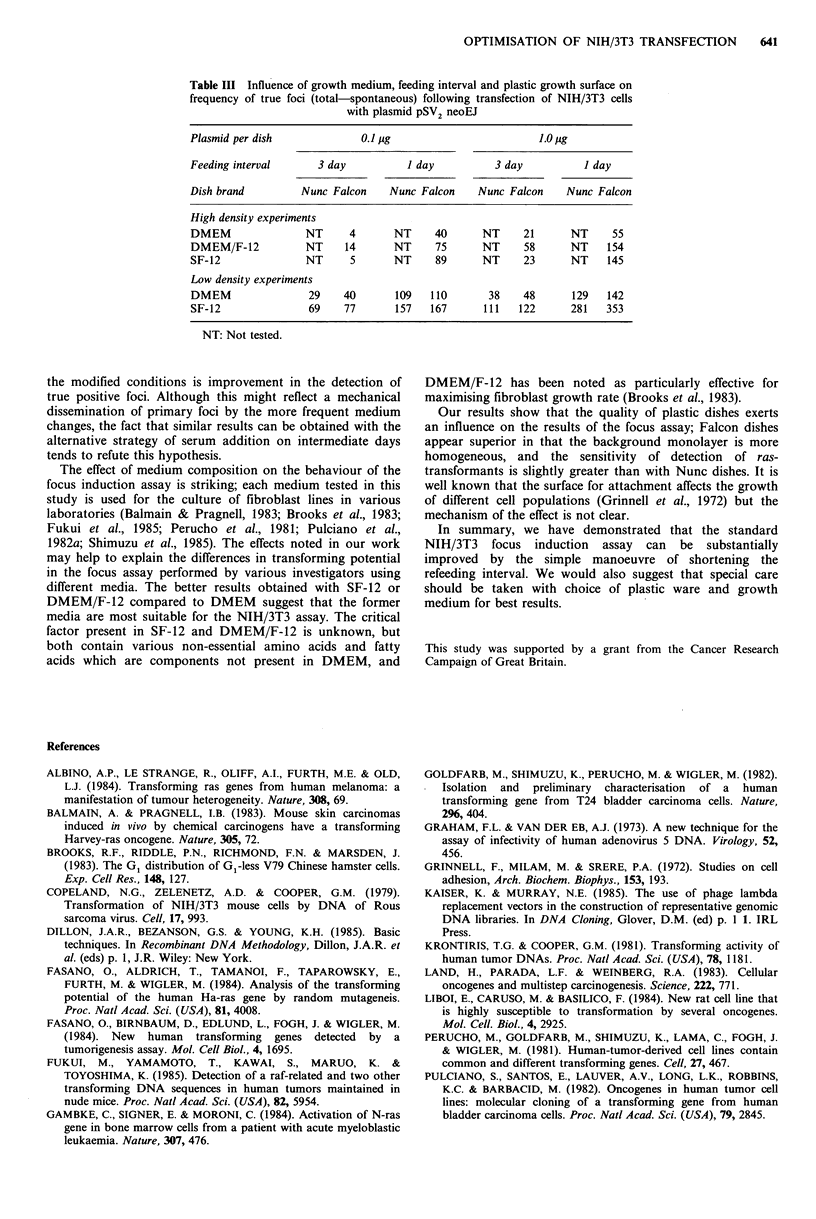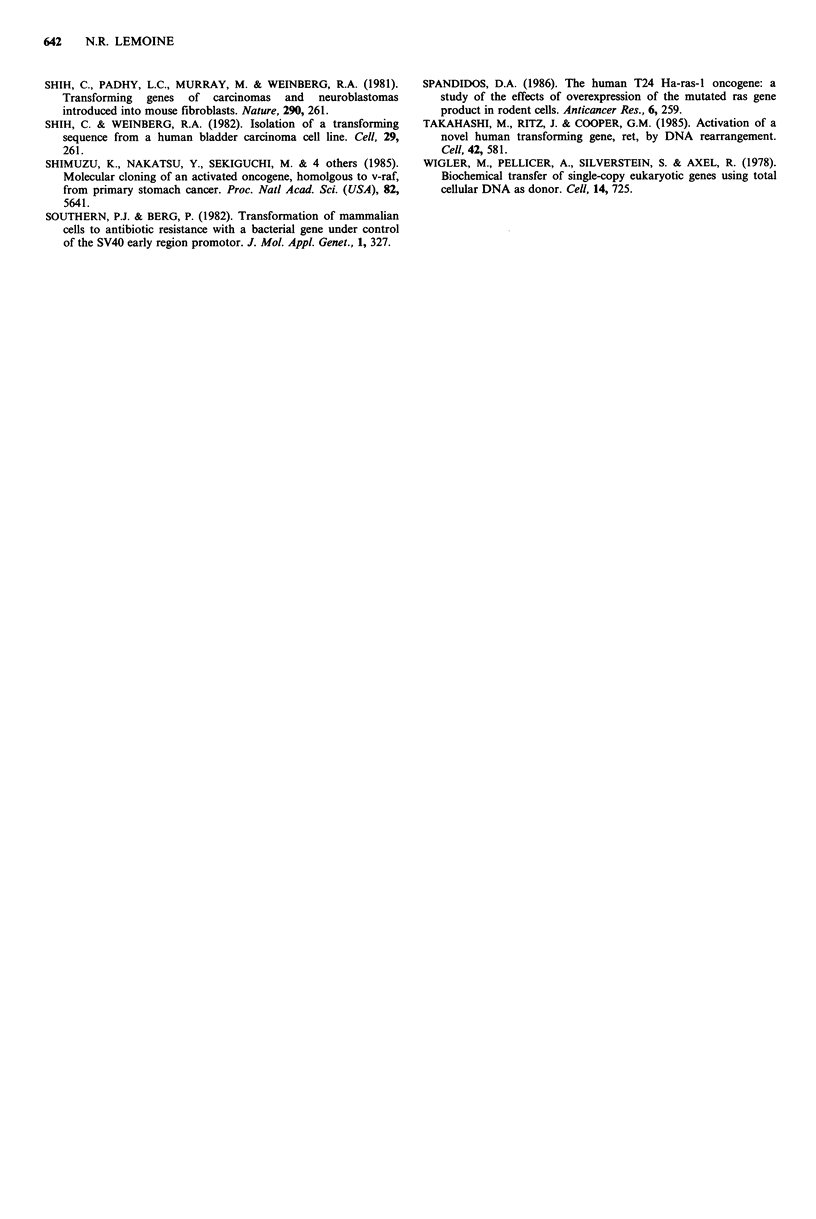# Optimisation of conditions for detection of activated oncogenes by transfection of NIH 3T3 cells.

**DOI:** 10.1038/bjc.1987.129

**Published:** 1987-06

**Authors:** N. R. Lemoine, V. Wynford-Thomas, D. Wynford-Thomas


*Br. J. Cancer* (1987), **55**, 639–642 © The Macmillan Press Ltd., 1987

SHORT COMMUNICATION

### Optimisation of conditions for detection of activated oncogenes by transfection of NIH 3T3 cells

N.R. Lemoine, V. Wynford-Thomas & D. Wynford-Thomas

*Cancer Biology Unit, Department of Pathology, University of Wales College of Medicine, Heath Park, Cardiff CF4 4XN, UK.*

NIH/3T3 cells have been widely used for the study of activated oncogenes in DNA derived from neoplastic tissue because this mouse fibroblast line is both an efficient recipient for DNA transfection and is susceptible to full transformation by a single activated oncogene, producing foci of transformed cells which can be detected directly by inspection of monolayer cultures (Goldfarb *et al.*, 1982; Krontiris & Cooper, 1981; Land *et al.*, 1983; Shih *et al.*, 1981). However, the popularity of NIH/3T3 cells has been tempered by their variable rate of spontaneous transformation, which has led to a search for alternative recipient cells for the analysis of activated oncogenes by focus assay (Liboi *et al.*, 1984) and the development of alternative assays, such as tumorigenesis in the nude mouse following cotransfection with a dominant selectable marker such as the *neo* gene (Fasano *et al.*, 1984).

The focus assay nevertheless has several potential advantages which, if it can be made reliable, would make it at least complementary to the cotransfection/nude mouse tumorigenicity assay. From a practical standpoint, an initial positive result is obtained more quickly (in 2 weeks, rather than 4–6 weeks for cotransfection and tumour growth in nude mice) and without the expense and possible inconvenience of using experimental animals. Since the focus assay depends on differential growth characteristics of the transformed cell, it produces a direct selection for the transformed phenotype which is maintained from the outset, and avoids the risk of losing the transforming sequence which might occur when selection is applied only for a cotransfected drug resistance gene.

The focus assay also allows for the possibility of cooperative intercellular effects from the majority, untransformed, population which may support the initial growth of the transformants; in the cotransfection procedure, these cells are killed from the outset, requiring the transformants to grow from very low density clones.

Picking individual foci in the simple focus assay allows the investigator to test *in vitro* parameters of transformation such as reduction in growth factor requirement and growth in soft agar while also growing up cells to mass culture for tumorigenicity testing and for DNA extraction if necessary. Cotransfection followed by injection of all surviving cells is essentially an all-or-none approach since no information can be retrieved if no tumour develops.

We therefore considered that the unique features of the focus assay justified its further study. Since, apart from the particular culture history of the subclone of NIH/3T3 cells used and batch to batch variations in the quality of serum (Balmain & Pragnell, 1983), little firm data existed on the source of variability in the standard NIH/3T3 assay, we set out to investigate systematically the optimum conditions. Spontaneous transformation is a greater problem in cultures grown at high cell density, and so our experiments used cultures initially seeded at relatively high density to ensure that any corrective measures were effective even under the most adverse conditions. The modifications to the assay were then applied to cultures with a lower initial seeding density, which are known to be more appropriate for the detection of transforming activity (Spandidos, 1986).

Correspondence: N.R. Lemoine.
Received 7 October 1986; and in revised form, 3 February 1987.

*Cell culture* NIH/3T3 cells (kindly provided by Dr C.J. Marshall, Institute of Cancer Research, London, UK), were routinely passaged in a 1:1 mixture of Dulbecco's modified Eagle's medium and Ham's F-12 (DMEM/F-12) with penicillin and streptomycin, and 10% calf serum, in a humified 5% $CO_2$ atmosphere.

Growth media employed for the transfection experiments were DMEM, or DMEM/F-12, or SF-12 (Flow); each was prepared with sodium bicarbonate at $2.2\,g\,l^{-1}$ (ideal buffering capacity for incubation in 5% $CO_2$) and pH adjusted to 7.4.

Transfection assays were performed in 90 mm Petri dishes obtained from either Nunc (Gibco) or Falcon (Becton Dickinson).

*DNA isolation* Plasmid $pSV_2$neoEJ consists of a 6.6 kb fragment of genomic DNA containing the activated Ha-*ras* from the EJ/T24 human bladder carcinoma cell line (Shih & Weinberg, 1982) cloned into the BamH1 site downstream from the *neo* gene (which confers resistance to $G_{418}$ in eukaryotes) and SV40 early region enhancer/promotor of $pSV_2$neo (Southern & Berg, 1982). Plasmid DNA was purified from Triton X-100 lysates by caesium chloride gradient ultracentrifugation (Dillon, Bezanson & Young, 1985).

Carrier DNA was extracted from normal human leucocytes by the method of Kaiser and Murray (1983); the average size of DNA obtained was greater than 80 kb.

*Transfection protocol* The technique employed was based on the methods of Graham and van der Eb (1973) and Wigler *et al.* (1978). On day 1, NIH/3T3 cells were plated at either (i) $1\times10^5$ cells per 90 mm dish ['low-density' experiments], or (ii) $5\times10^5$ cells per 90 mm dish ['high-density' experiments], in 10 ml of DMEM/F-12 with 10% calf serum.

On day 2, the dishes were refed with fresh growth medium (either DMEM or DMEM/F-12 or SF-12) with 10% calf serum 6 h before transfection. The calcium phosphate-DNA coprecipitate was prepared as follows: for each 90 mm dish, 0.1–1.0 $\mu$g closed circular plasmid DNA with carrier DNA to a total of 20 $\mu$g was diluted to 0.4 ml of 25 mM HEPES with 0.1 ml 1.25 M $CaCl_2$ and swirled to mix. This mixture was then added dropwise to 0.5 ml of HEPES-buffered saline (280 mM NaCl, 50 mM HEPES, 1.5 mM sodium phosphate, pH adjusted to 7.10) with gentle bubbling of a stream of air via a plastic pipette to ensure even mixing. The suspension was left undisturbed for 30 min at room temperature, and then 1 ml (20 $\mu$g DNA) was added to each 90 mm dish.

The dishes were incubated at 37°C for 16 h, when the suspension was removed and the dishes washed twice with

Tris-buffered saline (20 mM Tris-HCl pH 7.4, 137 mM NaCl, 5 mM KCl) Fresh growth medium (either DMEM or DMEM/F-12 or SF-12) with 10% calf serum was added and incubation continued for a further 24 h, when the medium was replaced with growth medium (DMEM or DMEM/F-12 or SF-12) supplemented with calf serum at concentrations ranging from 2% to 20% (see below).

The dishes were refed with fresh medium either every 3 days as in most previous studies using the focus induction assay (Albino *et al.*, 1984; Copeland *et al.*, 1979; Fasano *et al.*, 1984; Fukui *et al.*, 1985; Gambke *et al.*, 1984; Perucho *et al.*, 1981; Pulciano *et al.*, 1982a, b; Shimuzu *et al.*, 1985; Takahashi *et al.*, 1985), or every day. A pilot study showed that there was no significant difference between regimes of 3 days interval and 2 days interval.

Foci were scored at 21 days after transfection.

*Frequency of spontaneous transformation* In cultures treated by mock transfection with calcium phosphate coprecipitate containing no DNA and maintained in the standard conditions (5% calf serum, 3 day refeeding interval) for 21 days an unacceptable frequency of spontaneous transformants was noted (Table I). As expected, the problem was of greater magnitude in 'high-density' cultures, but also significant in the dishes seeded initially with $1 \times 10^5$ cells. In addition, the background monolayer became fragile and ragged (particularly in Nunc dishes) with cycles of cell death, most marked immediately before refeeding, followed by rapid regrowth after addition of fresh medium. The fluctuation in growth factor concentration over 3 day cycles favours the emergence of spontaneous foci of cells with a pseudotransformed morphology.

**Table I** Influence of calf serum concentration on maximal cell density and frequency of spontaneous foci in the NIH/3T3 focus induction assay using a 3 day refeeding interval in dishes seeded with $1 \times 10^5$ cells

| *Serum concentration* (%) | *Maximal cell density* ($+10^4\,cm^{-2}$) | *Spontaneous foci* (*per 90 mm dish*) |
|---|---|---|
| 2 | —[a] | 0 |
| 5 | 4.4 | 20 |
| 7 | 8.2 | 55 |
| 10 | 11.2 | 90 |
| 20 | 14.1 | >100 |

[a]A monolayer could not be sustained by 2% calf serum.

We attempted to overcome the problem by varying four parameters:

(i) *Serum concentration* Concentrations of less than 5% calf serum could not support a confluent monolayer, while increase of the concentration above 5% merely produced an increase in cell density and a higher incidence of spontaneous transformations. With concentrations above 10% calf serum the majority of cells showed a pseudotransformed morphology (Table I). (ii) *Refeeding interval* To test the hypothesis that the generation of spontaneous 'pseudo-transformed' foci was the result of wide fluctuations in growth factor concentrations, the refeeding interval was shortened from 3 days to 1 day, which would be expected to smooth the peaks and troughs of labile growth factor concentration. The result was a dramatic improvement in the quality of the backgriund with 5% calf serum: a quiescent flat monolayer without spontaneous foci even at 21 days was reproducibly obtained, regardless of the batch of serum used (Table II). (iii) *Growth medium* Because of the variety of growth media employed for the focus assay in different laboratories, we tested the effect of medium composition in our experiments. A significant difference was noted between dishes maintained in DMEM/F-12 or SF-12 and those maintained in DMEM. With the standard 3 day refeeding regime DMEM produced a higher population density and more spontaneous foci than DMEM/F-12 or SF-12; with the 1 day regime, spontaneous foci were not seen in any growth medium (Table II). (iv) *Plastic surface* We tested the influence of two widely-used brands of tissue culture dishes (Nunc dishes from Gibco, Falcon from Becton Dickinson) and found that the frequency of spontaneous foci was similar for each brand when cells were seeded at 'high density', but somewhat lower in Falcon dishes when cells were seeded at the 'low density' commonly used for the focus induction assay (Table II).

*Efficiency of the focus assay* Having optimised conditions to produce a minimum false positive rate (even under the adverse conditions of high initial seeding density), we then tested the sensitivity of the modified assay for detection of activated Ha-*ras*. Table III summarises the influence of the tested variables on the frequency of *ras*-transformed foci at 21 days.

The foci appeared on day 9 after transfection when DMEM/F-12 or SF-12 growth media were used, but not until day 14 with DMEM. The induced foci had the typical morphology of *ras*-transformants in all media tested, consisting of refractile cells growing at high density in a criss-cross arrangement with a notable proportion of giant cells.

The transformation efficiency of $pSv_2neoEJ$ is maximum in the modified assay conditions, reaching a peak of 1,670 foci $\mu g^{-1}$ when 0.1 $\mu g$ of plasmid was transfected into each $1 \times 10^5$ cells in Falcon dishes in SF-12 medium changed every day. This calculated value is similar to that reported with cloned Ha-*ras*-1 gene by other workers (Goldfarb *et al.*, 1982; Shih & Weinberg, 1982). (It should be noted that calculated transformation efficiency varies in a non-linear manner with dose of recombinant gene.)

Our results show that shortening the refeeding interval to 1 day produces the optimum background for the NIH/3T3 focus induction assay, with a zero false positive rate. A similar result can also be achieved with the standard 3 day regime if 0.5 ml of serum is added to each 90 mm dish on the days between medium changes. The particular batch of serum does not influence this effect. An additional benefit of

**Table II** Influence of initial plating density, growth medium, feeding interval and plastic growth surface on frequency of spontaneous foci

| | *Incidence of spontaneous foci per 90 mm dish* | | | | | | | |
|---|---|---|---|---|---|---|---|---|
| | *Low density experiments* | | | | *High density experiments* | | | |
| *Feeding interval* | *3 days* | | *1 day* | | *3 days* | | *1 day* | |
| *Dish brand* | *Nunc* | *Falcon* | *Nunc* | *Falcon* | *Nunc* | *Falcon* | *Nunc* | *Falcon* |
| DMEM | 25 | 20 | 0 | 0 | 30 | 32 | 0 | 0 |
| DMEM/F-12 | NT | NT | NT | NT | 20 | 20 | 0 | 0 |
| SF-12 | 7 | 3 | 0 | 0 | 18 | 18 | 0 | 0 |

NT: Not tested.

**Table III** Influence of growth medium, feeding interval and plastic growth surface on frequency of true foci (total—spontaneous) following transfection of NIH/3T3 cells with plasmid $pSV_2$ neoEJ

| *Plasmid per dish* | *0.1 µg* | | | | *1.0 µg* | | | |
|---|---|---|---|---|---|---|---|---|
| *Feeding interval* | *3 day* | | *1 day* | | *3 day* | | *1 day* | |
| *Dish brand* | *Nunc* | *Falcon* | *Nunc* | *Falcon* | *Nunc* | *Falcon* | *Nunc* | *Falcon* |
| *High density experiments* | | | | | | | | |
| DMEM | NT | 4 | NT | 40 | NT | 21 | NT | 55 |
| DMEM/F-12 | NT | 14 | NT | 75 | NT | 58 | NT | 154 |
| SF-12 | NT | 5 | NT | 89 | NT | 23 | NT | 145 |
| *Low density experiments* | | | | | | | | |
| DMEM | 29 | 40 | 109 | 110 | 38 | 48 | 129 | 142 |
| SF-12 | 69 | 77 | 157 | 167 | 111 | 122 | 281 | 353 |

NT: Not tested.

the modified conditions is improvement in the detection of true positive foci. Although this might reflect a mechanical dissemination of primary foci by the more frequent medium changes, the fact that similar results can be obtained with the alternative strategy of serum addition on intermediate days tends to refute this hypothesis.

The effect of medium composition on the behaviour of the focus induction assay is striking; each medium tested in this study is used for the culture of fibroblast lines in various laboratories (Balmain & Pragnell, 1983; Brooks *et al.*, 1983; Fukui *et al.*, 1985; Perucho *et al.*, 1981; Pulciano *et al.*, 1982*a*; Shimuzu *et al.*, 1985). The effects noted in our work may help to explain the differences in transforming potential in the focus assay performed by various investigators using different media. The better results obtained with SF-12 or DMEM/F-12 compared to DMEM suggest that the former media are most suitable for the NIH/3T3 assay. The critical factor present in SF-12 and DMEM/F-12 is unknown, but both contain various non-essential amino acids and fatty acids which are components not present in DMEM, and DMEM/F-12 has been noted as particularly effective for maximising fibroblast growth rate (Brooks *et al.*, 1983).

Our results show that the quality of plastic dishes exerts an influence on the results of the focus assay; Falcon dishes appear superior in that the background monolayer is more homogeneous, and the sensitivity of detection of *ras*-transformants is slightly greater than with Nunc dishes. It is well known that the surface for attachment affects the growth of different cell populations (Grinnell *et al.*, 1972) but the mechanism of the effect is not clear.

In summary, we have demonstrated that the standard NIH/3T3 focus induction assay can be substantially improved by the simple manoeuvre of shortening the refeeding interval. We would also suggest that special care should be taken with choice of plastic ware and growth medium for best results.

This study was supported by a grant from the Cancer Research Campaign of Great Britain.

**References**

ALBINO, A.P., LE STRANGE, R., OLIFF, A.I., FURTH, M.E. & OLD, L.J. (1984). Transforming ras genes from human melanoma: a manifestation of tumour heterogeneity. *Nature*, **308**, 69.

BALMAIN, A. & PRAGNELL, I.B. (1983). Mouse skin carcinomas induced *in vivo* by chemical carcinogens have a transforming Harvey-ras oncogene. *Nature*, **305**, 72.

BROOKS, R.F., RIDDLE, P.N., RICHMOND, F.N. & MARSDEN, J. (1983). The $G_1$ distribution of $G_1$-less V79 Chinese hamster cells. *Exp. Cell Res.*, **148**, 127.

COPELAND, N.G., ZELENETZ, A.D. & COOPER, G.M. (1979). Transformation of NIH/3T3 mouse cells by DNA of Rous sarcoma virus. *Cell*, **17**, 993.

DILLON, J.A.R., BEZANSON, G.S. & YOUNG, K.H. (1985). Basic techniques. In *Recombinant DNA Methodology*, Dillon, J.A.R. *et al.* (eds) p. 1, J.R. Wiley: New York.

FASANO, O., ALDRICH, T., TAMANOI, F., TAPAROWSKY, E., FURTH, M. & WIGLER, M. (1984). Analysis of the transforming potential of the human Ha-ras gene by random mutageneis. *Proc. Natl Acad. Sci.* (*USA*), **81**, 4008.

FASANO, O., BIRNBAUM, D., EDLUND, L., FOGH, J. & WIGLER, M. (1984). New human transforming genes detected by a tumorigenesis assay. *Mol. Cell Biol.*, **4**, 1695.

FUKUI, M., YAMAMOTO, T., KAWAI, S., MARUO, K. & TOYOSHIMA, K. (1985). Detection of a raf-related and two other transforming DNA sequences in human tumors maintained in nude mice. *Proc. Natl Acad. Sci.* (*USA*), **82**, 5954.

GAMBKE, C., SIGNER, E. & MORONI, C. (1984). Activation of N-ras gene in bone marrow cells from a patient with acute myeloblastic leukaemia. *Nature*, **307**, 476.

GOLDFARB, M., SHIMUZU, K., PERUCHO, M. & WIGLER, M. (1982). Isolation and preliminary characterisation of a human transforming gene from T24 bladder carcinoma cells. *Nature*, **296**, 404.

GRAHAM, F.L. & VAN DER EB, A.J. (1973). A new technique for the assay of infectivity of human adenovirus 5 DNA. *Virology*, **52**, 456.

GRINNELL, F., MILAM, M. & SRERE, P.A. (1972). Studies on cell adhesion, *Arch. Biochem. Biophys.*, **153**, 193.

KAISER, K. & MURRAY, N.E. (1985). The use of phage lambda replacement vectors in the construction of representative genomic DNA libraries. In *DNA Cloning*, Glover, D.M. (ed) p. 1 **1**. IRL Press.

KRONTIRIS, T.G. & COOPER, G.M. (1981). Transforming activity of human tumor DNAs. *Proc. Natl Acad. Sci.* (*USA*), **78**, 1181.

LAND, H., PARADA, L.F. & WEINBERG, R.A. (1983). Cellular oncogenes and multistep carcinogenesis. *Science*, **222**, 771.

LIBOI, E., CARUSO, M. & BASILICO, F. (1984). New rat cell line that is highly susceptible to transformation by several oncogenes. *Mol. Cell. Biol.*, **4**, 2925.

PERUCHO, M., GOLDFARB, M., SHIMUZU, K., LAMA, C., FOGH, J. & WIGLER, M. (1981). Human-tumor-derived cell lines contain common and different transforming genes. *Cell*, **27**, 467.

PULCIANO, S., SANTOS, E., LAUVER, A.V., LONG, L.K., ROBBINS, K.C. & BARBACID, M. (1982). Oncogenes in human tumor cell lines: molecular cloning of a transforming gene from human bladder carcinoma cells. *Proc. Natl Acad. Sci.* (*USA*), **79**, 2845.

SHIH, C., PADHY, L.C., MURRAY, M. & WEINBERG, R.A. (1981). Transforming genes of carcinomas and neuroblastomas introduced into mouse fibroblasts. *Nature*, **290**, 261.

SHIH, C. & WEINBERG, R.A. (1982). Isolation of a transforming sequence from a human bladder carcinoma cell line. *Cell*, **29**, 261.

SHIMUZU, K., NAKATSU, Y., SEKIGUCHI, M. & 4 others (1985). Molecular cloning of an activated oncogene, homolgous to v-raf, from primary stomach cancer. *Proc. Natl Acad. Sci.* (*USA*), **82**, 5641.

SOUTHERN, P.J. & BERG, P. (1982). Transformation of mammalian cells to antibiotic resistance with a bacterial gene under control of the SV40 early region promotor. *J. Mol. Appl. Genet.*, **1**, 327.

SPANDIDOS, D.A. (1986). The human T24 Ha-ras-1 oncogene: a study of the effects of overexpression of the mutated ras gene product in rodent cells. *Anticancer Res.*, **6**, 259.

TAKAHASHI, M., RITZ, J. & COOPER, G.M. (1985). Activation of a novel human transforming gene, ret, by DNA rearrangement. *Cell*, **42**, 581.

WIGLER, M., PELLICER, A., SILVERSTEIN, S. & AXEL, R. (1978). Biochemical transfer of single-copy eukaryotic genes using total cellular DNA as donor. *Cell*, **14**, 725.